# The Voltage-Gated Sodium Channel Beta4 Subunit Maintains Epithelial Phenotype in Mammary Cells

**DOI:** 10.3390/cells10071624

**Published:** 2021-06-29

**Authors:** Adélaïde Doray, Roxane Lemoine, Marc Severin, Stéphanie Chadet, Osbaldo Lopez-Charcas, Audrey Héraud, Christophe Baron, Pierre Besson, Arnaud Monteil, Stine Falsig Pedersen, Sébastien Roger

**Affiliations:** 1 Transplantation, Immunologie et Inflammation T2I-EA 4245, Université de Tours, 37044 Tours, France; adelaide.doray@etu.univ-tours.fr (A.D.); roxane.lemoine@univ-tours.fr (R.L.); stephanie.chadet@univ-tours.fr (S.C.); osbaldo.lopez@univ-tours.fr (O.L.-C.); audrey.heraud@univ-tours.fr (A.H.); christophe.baron@univ-tours.fr (C.B.); pierre.besson@univ-tours.fr (P.B.); 2 Section for Cell Biology and Physiology, Department of Biology, Faculty of Science, University of Copenhagen, 2100 Copenhagen, Denmark; marc.severin@bio.ku.dk (M.S.); sfpedersen@bio.ku.dk (S.F.P.); 3 Institut de Génomique Fonctionnelle, University of Montpellier, CNRS UMR 5203, INSERM U1191, 34094 Montpellier, France; arnaud.monteil@igf.cnrs.fr; 4 Institut Universitaire de France (IUF), 75231 Paris, France

**Keywords:** Na_V_β4, epithelial phenotype, β-catenin, epithelial-to-mesenchymal transition, mammary cells

## Abstract

The *SCN4B* gene, coding for the Na_V_β4 subunit of voltage-gated sodium channels, was recently found to be expressed in normal epithelial cells and down-regulated in several cancers. However, its function in normal epithelial cells has not been characterized. In this study, we demonstrated that reducing Na_V_β4 expression in MCF10A non-cancer mammary epithelial cells generated important morphological changes observed both in two-dimensional cultures and in three-dimensional cysts. Most notably, the loss of Na_V_β4 induced a complete loss of epithelial organisation in cysts and increased proteolytic activity towards the extracellular matrix. Loss of epithelial morphology was associated with an increased degradation of β-catenin, reduced E-cadherin expression and induction of mesenchymal markers N-cadherin, vimentin, and α-SMA expression. Overall, our results suggest that Navβ4 may participate in the maintenance of the epithelial phenotype in mammary cells and that its downregulation might be a determining step in early carcinogenesis.

## 1. Introduction

Voltage-gated sodium channels β (Na_V_β) proteins, encoded by *SCNxB* genes, define a family of four transmembrane proteins with a short C-terminal intracellular domain and a large N-terminal immunoglobulin (Ig)-like extracellular domain [1]. These proteins were initially characterized as auxiliary subunits of sodium channels [2]. Indeed, they were isolated along with pore-forming voltage-gated sodium channel (Na_V_α) isoforms, which they interact with through covalent or non-covalent associations [3,4,5,6]. Hence, Na_V_β proteins regulate Na_V_α membrane trafficking as well as their biophysical [7] and pharmacological properties [8,9,10]. Along with these roles as sodium channel activity modulators, Na_V_β proteins have also been proven to conduct other specific cellular functions in excitable cells [11], such as an important role as cell adhesion molecules (CAMs), allowing for both trans-homophilic and trans-heterophilic cell–cell and cell–matrix adhesions in cells expressing Na_V_α isoforms [12]. Therefore, the expression and roles of Na_V_β are best known in excitable cells, such as neurons, in which they participate in the regulation of Na^+^ influx but also control neurite outgrowth, axonal fasciculation and interaction with glial cells [13].

The most recently identified Na_V_β4 subunit, encoded by the *SCN4B* gene, is the least characterized member of the family. It was initially demonstrated to be expressed in the nervous system (dorsal root ganglia, spinal cord, and restricted areas or nuclei in the brain), in skeletal and cardiac muscle cells [14]. It shares sequence similarity with the Na_V_β2 subunit and engages in covalent interactions with Na_V_α in the extracellular Ig domain [6,14]. Na_V_β4 was demonstrated to control Na_V_α activity and particularly the generation of resurgent [15,16,17] or persistent [18,19] sodium currents in neurons. Na_V_β4 was also identified to participate in cell–cell adhesion [20] and neurite extension [21]. Evidence is shown that Na_V_β4 dysregulation is involved in epilepsy [22], in Rett syndrome [23], and mutations in the *SCN4B* gene have been linked to cardiac arrythmia [24,25,26,27] and sudden death syndromes [28].

Recently, we identified a critical role for Na_V_β4 in cancer progression [29]. Specifically, we have shown that Na_V_β4 was strongly expressed in normal epithelial cells and tissues from breast, colon, rectum, lung, and prostate but consistently downregulated in cancer samples, to be almost absent in high-grade primary and metastatic tumours [29]. In mammary cancer cells, reducing Na_V_β4 expression potentiated cell migration and invasiveness through the acquisition of a hybrid mesenchymal–amoeboid aggressive phenotype, which resulted in an increase in mammary tumour growth and a higher metastatic colonisation. This effect was independent of the Na_V_α channel activity [29]. Later, similar results were obtained in cervical cancer cells [30], and the preserved expression of *SCN4B* in papillary thyroid cancer was proposed to be a favourable indicator of a recurrence-free survival [31]. It was therefore suggested that the *SCN4B* gene might be considered as a metastasis suppressor gene [29]. Nevertheless, the function of Na_V_β4 in normal epithelial cells is not known. We therefore hypothesized that Na_V_β4 is important for epithelial phenotype. To test this, we explored the consequences of reducing Na_V_β4 expression in non-cancer MCF10A mammary cells. We showed that knocking-down Na_V_β4 induced a loss of epithelial phenotype that was reminiscent of early stages of carcinogenesis.

## 2. Material and Methods

Bioinformatic analyses—Gene expression data were obtained from The Cancer Genome Atlas (TCGA) and Genotype-Tissue Expression (GTEx) databases using the UCSC Xena Browser (https://xenabrowser.net accessed on 19 December 2020). The IlluminaHiSeq (log2-normalized_count+1) files were downloaded from the “TCGA Breast Cancer (TCGA-BRCA)” cohort in order to compare expressions between adjacent non-tumoral tissues and primary tumour. From the “TCGA TARGET GTEx” cohort, the RSEM norm_count (log2-normalized_count+1) files were downloaded in order to compare expressions between adjacent non-tumoral tissues, primary tumour and metastases.

Inhibitors and chemicals—The proteasome inhibitor MG132 as well as all chemicals were purchased from Sigma-Aldrich (St. Quentin Fallavier, France). Fluorescent probes DQ™-Gelatin and Hoechst 33342 were purchased from Invitrogen (Villebon sur Yvette, France), and Phalloidin-AF594 from Cell Signalling Technology (Ozyme, Saint-Cyr-L’Ecole, France). ProLong^®^ Gold Antifade Mountant containing DAPI was purchased from Invitrogen (Villebon sur Yvette, France).

Cells and cell culture—The human breast cancer cell line MDA-MB-231 and non-cancer mammary epithelial MCF10A cells were acquired from the American Type Culture Collection (ATCC Manassas, Virginia), through LGC Standards (Molsheim, France), and were cultivated at 37 °C in a humidified 5% CO_2_ incubator. MDA-MB-231 cells were cultivated in Dulbecco’s modified Eagle’s medium (DMEM) supplemented with 5% foetal calf serum (FCS). The immortalized non-cancer mammary epithelial MCF10A cells were cultured in DMEM/Ham’s F-12, 1:1 mix containing 5% horse serum (Dutscher, Bernolsheim, France), 10 µg/mL insulin, 20 ng/mL epidermal growth factor, 0.5 µg/mL hydrocortisone, and 100 ng/mL cholera toxin. A stable MCF10A cell line knocked-down for the expression of the *SCN4B* gene, coding for Na_V_β4, was generated using the CRISPR/Cas9 technique, as previously described [32] by transfection with the *SCN4B* Double Nickase Plasmid (sc-411001, Santa Cruz, France). Transfection was performed using Lipofectamine 2000 (Invitrogen, Villebon sur Yvette, France). Selection of a stable cell line called “MCF10A Crβ4” was performed using 10 µg/mL puromycin and was compared with a control cell line, thereafter called “MCF10A CTL”, which received all treatments similarly to MCF10A Crβ4 but was not transfected with the guide RNA sequence specific for excision of the *SCN4B* gene. Efficiency of the CRISPR-mediated knock-down was assessed by western blotting, and the stability of cells was followed for a minimal duration of 6 weeks. Mycoplasma contamination tests were performed routinely (Lonza, MycoAlert™ Mycoplasma Detection Kit, Levallois-Perret, France).

Small interfering RNA transfection—MCF10A mammary epithelial cells were transfected with siRNA directed against human *SCN4B* mRNA (siβ4) or scramble siRNA as a control (siCTL), both of which were purchased from ON-TARGETplus siRNA (Horizon Discovery, Cambridge, UK). Cells were transfected using Pepmute™ siRNA Transfection Reagent (SignaGen laboratories, Rockville, MD, USA). Experiments were performed 24–48 h after transfection and efficacy of silencing was assessed by western blotting.

Overexpression of *SCN4B* gene—MCF10A Crβ4 cells were transfected with 2 µg of a pcDNA3.1(+) plasmid containing the *SCN4B* gene (pc*SCN4B*, Synbio Technologies, Monmouth Junction, NJ, USA) or with a pcDNA3.1(+) empty vector as a control. Transfection was realized using Lipofectamine 2000 (Invitrogen, France).

RNA extraction, reverse transcription (RT) and quantitative-polymerase chain reaction (qPCR)—Total RNA was extracted using TRIzol™ Reagent (Invitrogen, Villebon sur Yvette, France), quantified by measuring absorbance at 260 nm using Nanodrop 2000™ (Thermofisher, Illkirch, France) and reverse-transcribed with the PrimeScript™ RT Reagent Kit (Takara Bio Group, Saint-Germain-en-Laye, France). Quantitative PCR were performed using SYBR qPCR Premix Ex Taq (Takara Bio Group, Saint-Germain-en-Laye, France) and LightCycler 384 wells (Roche, Boulogne-Billancourt, France). Control gene was *HPRT1* (Hypoxanthine Phosphoribosyltransferase 1). All primers sequences and corresponding efficiencies are described in Supplementary Table S1.

Cell viability—Cell viability was evaluated by the tetrazolium salt assay (MTT) as already described [33]. Briefly, cells were seeded in different densities indicated in the figure and grew for 4 days at 37 °C and 5% CO_2_ in their normal culture medium. Cell viability was measured after incubation with 0.5 mg/mL MTT for 60 min at 37 °C by measuring the absorbance at 540 nm.

Cell proliferation—Cells were seeded at a density of 2 × 10^5^ in wells of a 6-well plate. Then, 72 h after seeding, medium was removed, and cells were incubated in a fresh culture medium containing 10 µM 5-ethynyl-2′deoxyuridine (EdU). Cells were then washed in phosphate-buffered saline (PBS), trypsinized, and the incorporation of EdU was monitored by flow cytometry (BD FACS Canto, Becton Dickinson, Le pont de Claix, France) using the Click-iT™ Plus EdU Alexa Fluor488 kit (Invitrogen, Villebon sur Yvette, France).

Cysts production and analyses—A 40 µL layer of Geltrex^®^ (Sigma-Aldrich, St. Quentin Fallavier, France) was added into the wells of Nunc™ Lab-Tek™ II Chamber Slide™ (Thermo Scientific, Illkirch, France), mimicking the extracellular matrix. The chamber slide was incubated at 37 °C for 20 min. MCF10A CTL or MCF10A Crβ4 cell suspensions were prepared in medium containing 25 µL/mL Geltrex^®^ and seeded at 10,000 cells per 400 µL in the chamber slide wells. The culture medium of cells was replaced every 2 days by 400 µL containing 25 µL/mL Geltrex^®^. Pictures of cysts were taken in bright field before changing medium (Invitrogen EVOS M7000, Thermofisher, Illkirch, France) in order to monitor growth and circularity of cysts. After 3 weeks of culture, cysts were washed twice in PBS, then fixed in 4% paraformaldehyde (PFA, Invitrogen, Villebon sur Yvette, France) for 30 min at room temperature. Cysts were washed three times for 10 min in 100 mM glycine solution, then permeabilised for 5 min in a 0.5% Triton X-100 solution (Sigma-Aldrich, St. Quentin Fallavier, France) and washed three times for 10 min with PBS. Unspecific site blocking was be realized by incubating for 1h in 5% BSA solution. Primary antibodies in 150 µL of 5% BSA solution were added to the wells and incubated at 4 °C overnight. Wells were washed twice for 10 min in PBS, then 150 µL of fluorescent secondary antibodies were added for 1h at room temperature. Hoechst 33342 (1/1000, Invitrogen, Villebon sur Yvette, France) was used to visualize cell nuclei and Phalloidin-AF594 (Invitrogen, Villebon sur Yvette, France) to visualize the F-actin network. Wells were finally washed four times with PBS, and micrographs were obtained using confocal microscopy using a 20× objective (LEICA SP8 STED, Nanterre, France). Primary antibodies used were dedicated to identifying β-catenin (Cell Signalling Technology D10A8, 1/200, Ozyme, Saint-Cyr-L’Ecole, France), E-cadherin (Invitrogen 13-1700, 1/1000, Invitrogen, Villebon sur Yvette, France), Vimentin (Abcam ab92547, 1/100, Paris, France). Growth and circularity of cysts were analysed using the ImageJ software (https://imagej.nih.gov/ij/ accessed on 19 December 2020).

Western Blotting—Cells were washed with PBS and lysed in presence of a lysis buffer (50 mM Tris, pH7, 100 mM NaCl, 5 mM MgCl_2_, 10% glycerol, 1 mM EDTA) containing 5% sodium dodecyl sulphate (SDS) and protease inhibitors (S8830, Sigma-Aldrich, St. Quentin Fallavier, France). Western blotting experiments were performed according to standard protocols. Total protein concentrations were determined using the Pierce^®^ BCA Protein Assay Kit Thermoscientific (Fisher Scientific, Illkirch-Graffenstaden, France). Protein sample buffer was added, and the samples were heated at 95 °C for 5 min. Total protein samples were electrophoretically separated by SDS-polyacrylamide gel electrophoresis in 10% gels and then transferred to polyvinylidene fluoride membranes (Millipore, Merck, Molsheim, France). Na_V_β4 proteins were detected using anti-Na_V_β4 rabbit polyclonal primary antibodies (1/5000, HPA017293, Sigma-Aldrich, St. Quentin Fallavier, France). Other primary antibodies used were: rabbit monoclonal anti-β-catenin (Cell Signalling Technology D10A8, 1/2000, Ozyme, Saint-Cyr-L’Ecole, France), mouse monoclonal anti-E-cadherin (Cell Signalling Technology, 4A2, 1/2000, Ozyme, Saint-Cyr-L’Ecole, France), rabbit monoclonal anti-vimentin (Cell Signalling Technology, D21H3, 1/2000, Ozyme, Saint-Cyr-L’Ecole, France), rabbit monoclonal anti-αSMA (Cell Signalling Technology, D4K9N, 1/2000, Ozyme, Saint-Cyr-L’Ecole, France), rabbit monoclonal anti-N-cadherin (Cell Signalling Technology, D4R1H, 1/2000, Ozyme, Saint-Cyr-L’Ecole, France). Secondary horseradish peroxidase (HRP)-conjugated goat anti-mouse and goat anti-rabbit IgG antibodies were obtained from BioRad (1/5000, Marnes-la-Coquette, France) and from Jackson ImmunoResearch (1/1000 Interchim, Saint-Cyr-L’École, France), respectively. HSC70 protein was used as a sample loading control using anti-HSC70 mouse primary antibody at 1/10,000 (Santa-Cruz). In some conditions, a β-actin-HRP mouse monoclonal antibody (1/1000, SantaCruz) was used as a control for sample loading. Proteins were detected using electrochemiluminescence-plus kit (Pierce^®^ ECL Western Blotting Substrate, Fisher Scientific, Illkirch, France) and captured on CL-XPosure Films (Thermoscientific, Illkirch, France). Densitometric analyses were performed using the ImageJ software, and quantifications of proteins of interest are expressed relatively to that of the control protein used, either HSC70 or β-actin, before being relativized to the adequate control condition. Full uncropped blots are shown in Supplementary Figure S2.

Epifluorescence experiments—MCF10A CTL and Crβ4 cells were cultivated for 48 h in Lab-Tek™ chambers (Sigma-Aldrich, St. Quentin Fallavier, France). In some cases, chambers were coated with a layer of Geltrex™ (Fisher Scientific, Illkirch, France) containing 25 µg/mL of DQ-Gelatin 488 (Invitrogen, Villebon sur Yvette, France). Cells were fixed in 4% paraformaldehyde for 30 min and then incubated with 5% BSA for 30 min. F-actin was visualized after staining the cells with phalloidin-AF594 (1/200, Cell Signalling Technology, Ozyme, Saint-Cyr-L’Ecole, France) for 1 h. Slides were mounted using ProLong^®^ Gold Antifade Mountant with DAPI to visualize cell nuclei (Invitrogen, Villebon sur Yvette, France). Epifluorescence microscopy was performed with an EVOS M7000 microscope (Thermofisher, Illkirch, France). Images were analysed using the ImageJ software.

Data presentation and statistical analysis—Data are displayed as mean ± standard error of the mean (sem) when following a normal distribution, or as individual points centred by a diagram showing the median when not following a normal distribution. One-way ANOVA followed by a Dunn’s Multiple Comparison Tests, two-way ANOVA, Mann–Whitney rank sum tests, and paired Student *t*-tests were used to compare different conditions as indicated in the figure legends. Statistical significance is indicated as: *, *p* < 0.05; **, *p* < 0.01, and ***, *p* < 0.001, while “ns” stands for not statistically different.

Data availability—The authors declare that all other data supporting the findings of this study are available within the paper, and its supplementary information files or available from the authors upon request.

## 3. Results

**Figure 1:**
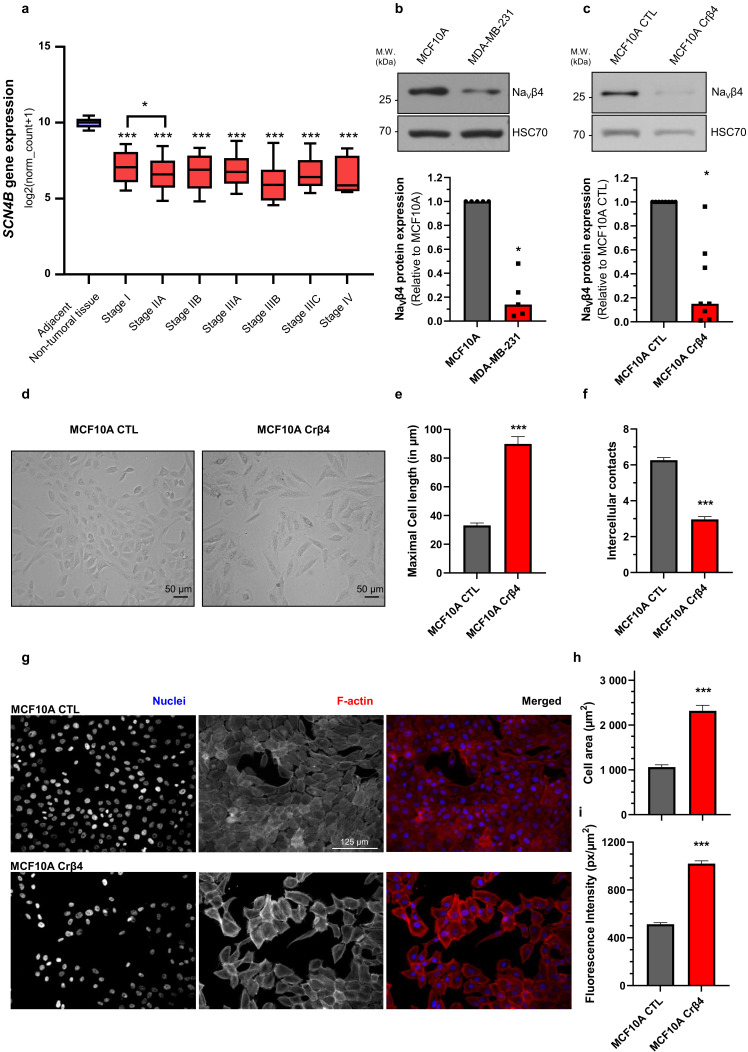
Na_V_β4 downregulation induces morphological changes in non-cancer mammary cells. (**a**) The expression level of the *SCN4B* gene, coding for Na_V_β4, was analysed from datasets from The Cancer Genome Atlas (http://cancergenome.nih.gov, accessed on 19 December 2020), from the US National Cancer Institute, in the non-tumoral adjacent tissue (n = 178), and in the different stages of primary breast tumours: I (n = 125), IIA (n = 243), IIB (n = 115), IIIA (n = 85), IIIB (n = 10), IIIC (n = 31), IV (n = 4). For each array, data were log2-transformed and centred to the median. ***, statistically different with *p* < 0.001 (Mann–Whitney rank sum test) when comparing with adjacent non-tumoral tissue; *, *p* < 0.05 when comparing Stage I with stage IIA. (**b**) Na_V_β4 protein expression level was assessed by western blotting in non-cancer MCF10A human mammary epithelial cells and in human breast cancer MDA-MB-231 cells. The upper section shows a WB representative of 5 independent experiments. HSC70 immunodetection was used as a loading control. The lower section shows a quantification of Na_V_β4 protein expression in the two cell lines expressed relatively to that of MCF10A. *, statistically different with *p* < 0.05 (Mann–Whitney rank sum test). (**c**) Na_V_β4 protein expression level was assessed by western blotting in control MCF10A cells and in cells stably knocked down for the expression of *SCN4B* gene (MCF10A Crβ4). The upper section shows a WB representative of 8 independent experiments. HSC70 immunodetection was used as a loading control. The lower section shows a quantification of Na_V_β4 protein expression in the two cell lines expressed relatively to that of MCF10A CTL (n = 8). *, statistically different with *p* < 0.05 (Mann–Whitney rank sum test). (**d**) Representative images of MCF10A CTL and MCF10A Crβ4 cells in phase contrast microscopy. Scale bar, 50 µm. (**e**) Maximal cell length (n = 31 MCF10A CTL and n = 20 MCF10A Crβ4) and, in (**f**), number of intercellular contacts per cell (n = 60 MCF10A CTL and n = 57 MCF10A Crβ4), assessed from images taken as in (**d**). Cells were randomly selected from pictures, and the number of joint cells was manually counted. ***, statistically different with *p* < 0.001 (Student’s *t*-test). (**g**) MCF10A CTL and MCF10A Crβ4 cells were stained for the identification of nuclei (DAPI, blue staining) and F-actin (phalloidin-594, red staining). Scale bar, 125 µm. (**h**) Mean cell area (n = 40 MCF10A CTL and n = 40 MCF10A Crβ4) and, in (**i**), F-actin fluorescence intensity per cell surface (n = 100 MCF10A CTL and n = 100 MCF10A Crβ4) were calculated from images taken as in (**g**). ***, statistically different with *p* < 0.001 (Student’s *t*-test).

Bioinformatic gene expression analyses, using The Cancer Genome Atlas (TCGA) and the UCSC Xena browser (https://xenabrowser.net accessed on 19 December 2020), confirmed initial studies [29] indicating that the *SCN4B* gene, coding for Na_V_β4, is significantly down-regulated in all breast cancer stages compared with adjacent non-tumoral breast tissues (Figure 1a). A lower expression of *SCN4B* was even identified in stage IIA compared with stage I (Figure 1a). In line with these initial results, Na_V_β4 protein expression was almost 10 times lower in the human mammary cancer MDA-MB-231 compared with the non-cancer MCF10A cells (Figure 1b). Importantly, this non-cancer, and non-tumorigenic, cell line was demonstrated not to express any functional pore-forming Na_V_ subunit [34,35], while human breast cancer cells express the Na_V_1.5 isoform which enhances invasive capacities [36,37]. In order to assess the potential role of Na_V_β4 in non-cancer mammary cells, we developed, using the CRISPR/Cas9 technique, a cell line derived from the parental MCF10A with a permanent knockdown of Na_V_β4, named MCF10A Crβ4, as well as a control cell line MCF10A CTL. The efficiency of the Na_V_β4 knock-down was verified by western blotting indicating a median reduction of protein expression of 85% (Figure 1c). Reduction of Na_V_β4 had important effects on the MCF10A cell morphology, as observed in conventional 2-dimensional cultures (Figure 1d). Particularly, MCF10A Crβ4 showed an elongated morphology characterised by an increased cell length as compared with MCF10A CTL (Figure 1e, 89.9 ± 5.1 vs. 33.1 ± 1.7 µm, respectively). Furthermore, while the MCF10A CTL cells showed a tendency for being closely associated in clusters, as epithelial cells do, the MCF10A Crβ4 cells were more scattered and established less intercellular interactions (Figure 1f). These morphological changes were also identified by epifluorescence imaging in fixed cells labelled to visualize cell nuclei and the F-actin network (Figure 1g). Image analysis demonstrated that MCF10A Crβ4 showed a greater cell surface than the MCF10A CTL cells (Figure 1h, 2318 ± 123 vs. 1062 ± 52 µm^2^, respectively). Interestingly, MCF10A Crβ4 also appeared to have a stronger intensity of F-actin labelling (Figure 1i), characterised by the presence of actin stress fibres (Figure 1g).

**Figure 2:**
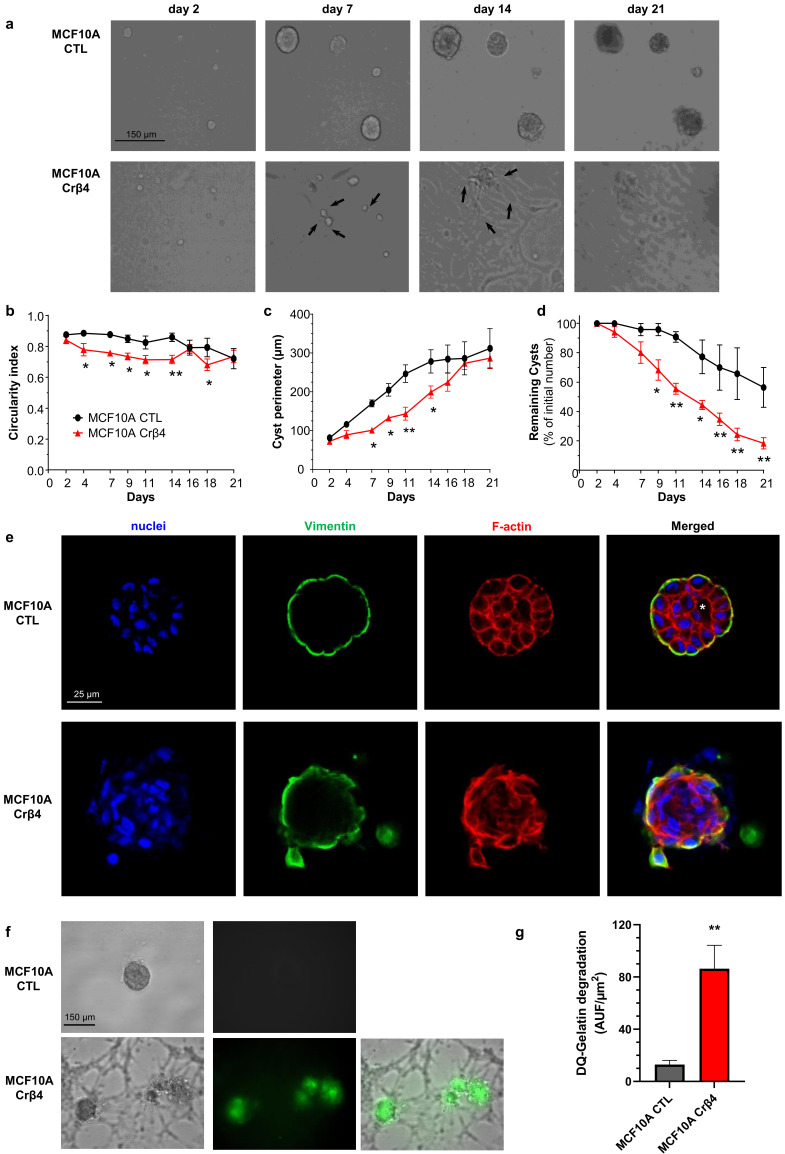
Na_V_β4 sustains epithelial polarity in 3-dimensional MCF10A cysts. (**a**) MCF10A CTL and MCF10A Crβ4 cells were cultivated as cysts for 21 days in a 3D matrix. Representative images at days 2, 7, 14, and 21 are shown. Black arrows show cells evading from cysts and invading the extracellular matrix. Scale bar, 150 µm. (**b**) The cyst circularity index was calculated from same cysts as in (**a**). Statistically different from MCF10A CTL: *, *p* < 0.05 (ANOVA test). (**c**) The perimeter of individual cysts was measured as a function of time from same cysts than in (**a**). Statistically different from MCF10A CTL: *, *p* < 0.05; **, *p* < 0.01 (ANOVA test). (**d**) The number of individualized cysts was assessed as a function of time and expressed relatively to the initial number at day 1 (n = 50 and 83 MCF10A CTL and MCF10A Crβ4 cysts, respectively, from 6 independent experiments). Statistically different from MCF10A CTL: *, *p* < 0.05; **, *p* < 0.01 (ANOVA test). (**e**) The MCF10A CTL and MCF10A Crβ4 cysts were then fixed and stained with Hoechst 33342 to visualize cell nuclei (blue staining), as well as with phalloidin-594 to visualize the F-actin network (red staining). Cysts were also immunostained with primary rabbit anti-vimentin antibodies and secondary AF488-coupled anti-rabbit antibodies (green staining). The white asterisk indicates the presence of a lumen inside the MCF10A CTL cysts. Representative images from 6 independent experiments. Scale bar, 25 µm. (**f**) Proteolytic activity of the cysts was assessed by including DQ-gelatin, which emits green fluorescence when degraded, in the 3D matrix. Representative images from 8 independent experiments. Scale bar, 150 µm. (**g**) An index of DQ-gelatin degradation was calculated from images in (**f**), from 16 individualized MCF10A CTL and 21 MCF10A Crβ4 cysts at day 7. Statistically different from the MCF10A CTL cysts: **, *p* < 0.01 (Student’s *t*-test).

**Figure 3:**
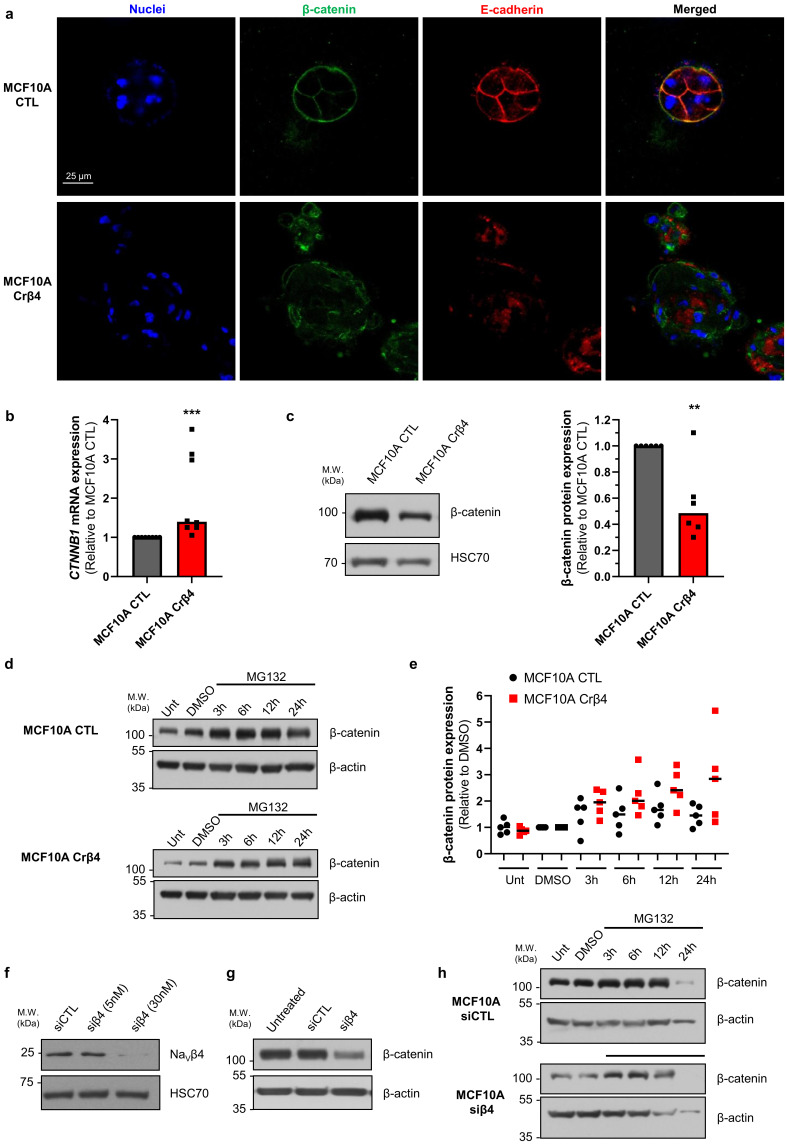
Na_V_β4 prevents β-catenin degradation. (**a**) MCF10A CTL and MCF10A Crβ4 cysts were stained with Hoechst 33342 to visualize cell nuclei and immunostained to identify β-catenin and E-cadherin. Representative images from 6 independent experiments. Scale bar, 25 µm. (**b**) Expression of the *CTNNB1* gene, coding for β-catenin, was analysed in the MCF10A CTL and MCF10A Crβ4 cells (n = 8 independent experiments). Statistically different from the MCF10A CTL cysts: ***, *p* < 0.001 (Mann–Whitney rank sum test). (**c**) Left, β-catenin protein expression level was assessed by western blotting in the MCF10A CTL and MCF10A Crβ4 cells. Representative WB from 6 independent experiments. Right, quantification of β-catenin protein expression in the MCF10A CTL and MCF10A Crβ4 cells (n = 6). **, statistically different with *p* < 0.01 (Mann–Whitney rank sum test). (**d**) β-catenin protein expression was assessed in untreated (Unt) MCF10A CTL and MCF10A Crβ4 cells or after the treatment with 10 µM MG132 for 3 h, 6 h, 12 h, or 24 h, or with the solvent DMSO at corresponding times. β-actin immunodetection was used as a loading control. (**e**) Quantification of β-catenin protein expression in the same conditions as in (**d**), from 5 independent experiments. Individual results are shown, centred by medians. Statistically different with *p* < 0.001 (two-way ANOVA). (**f**) Na_V_β4 protein expression was assessed by western blotting in MCF10A transfected with control “irrelevant” siRNA (siCTL) or with *SCN4B*-specific siRNA (siβ4) at 5 and 30 nM. (**g**) β-catenin protein expression was assessed in untreated MCF10A cells or in cells transfected with siCTL or siβ4 (30 nM). β-actin immunodetection was used as a loading control. Representative of 5 independent experiments. (**h**) β-catenin protein expression was assessed in the MCF10A cells transfected with siCTL or siβ4 (30 nM) after the treatment with 10 µM MG132 for 3 h, 6 h, 12 h, or 24 h, or with the solvent DMSO. β-actin immunodetection was used as a loading control.

**Figure 4:**
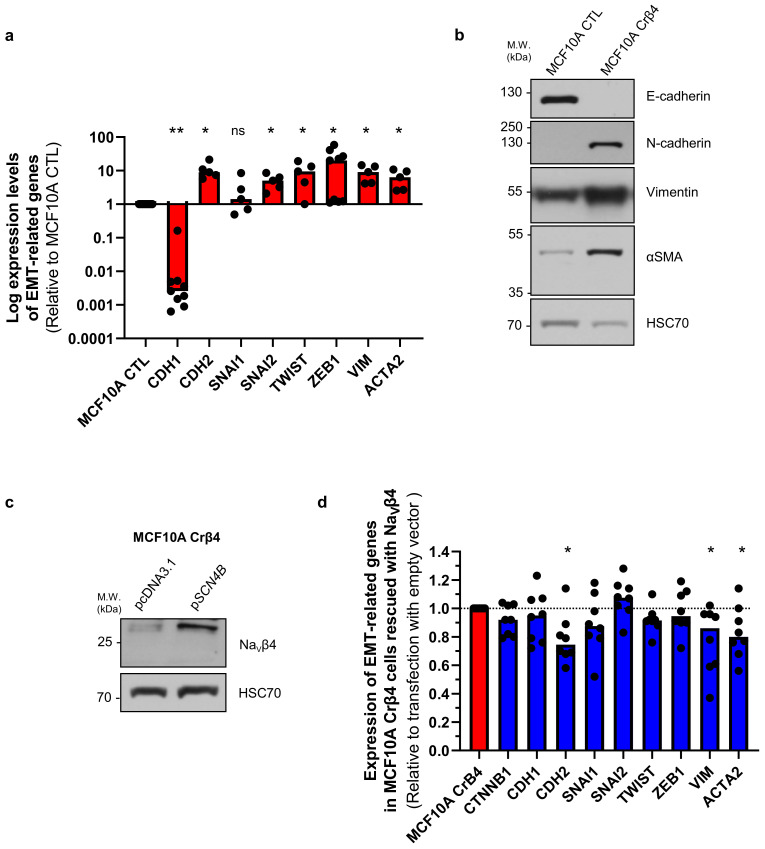
Na_V_β4 expression prevents mesenchymal transition in MCF10A epithelial mammary cells. (**a**) Expression of genes associated with either epithelial (*CDH1*) or mesenchymal (*CDH2*, *SNAI1*, *SNAI2*, *TWIST*, *ZEB1*, *VIM*, *ACTA2*) phenotype by RT-qPCR in MCF10A CTL and MCF10A Crβ4 cells (n = 5–9 independent experiments). Results are expressed relatively to those of the MCF10A CTL cells. “ns” stands for no statistical difference. Statistically different: *, *p* < 0.05; **, *p* < 0.01 (Mann–Whitney rank sum test). (**b**) Representative western blots showing the protein expression of E-cadherin, N-cadherin, vimentin, α-SMA in the MCF10A CTL and MCF10A Crβ4 cells. Immunodetection of HSC70 was used as a loading control (n = 4 independent experiments). (**c**) Na_V_β4 protein expression was assessed by western blotting in MCF10A Crβ4 cells transfected with empty pcDNA3.1 or with the *SCN4B* gene in the pcDNA3.1 vector. Representative of 8 independent experiments. (**d**) Expression of genes associated with either epithelial (*CTNNB1*, *CDH1*) or mesenchymal (*CDH2*, *SNAI1*, *SNAI2*, *TWIST*, *ZEB1*, *VIM*, *ACTA2*) phenotype by RT-qPCR in the MCF10A Crβ4 cells transfected with empty pcDNA3.1 or with the *SCN4B* gene in the pcDNA3.1 vector (n = 8 independent experiments). Results are expressed relatively to those of cells transfected with the empty vector. Statistically different: * *p* < 0.05 (Mann–Whitney rank sum test), otherwise no statistical difference.

These initial data suggested that the loss of Na_V_β4 expression affected epithelial cell phenotype and intercellular organization. Importantly, the loss of Na_V_β4 expression did not induce the expression of the *SCN5A* gene coding for Na_V_1.5 (Supplementary Figure S1a). We next investigated how the loss of Na_V_β4 affected cell polarisation in three dimensions, using a cyst forming assay in a 3D extracellular matrix [38]. We found that, in 3D cultures, the MCF10A CTL cells formed regular cysts with a general spheroid morphology (Figure 2a), characterized by a circularity index (calculated from pictures) approaching 0.9, maintained during the 3 weeks of the experiment (Figure 2b). The MCF10A CTL cysts demonstrated a regular growth that was slightly slowed after 14 days of culture (Figure 2c). After this time, the number of remaining individualized cysts tended to decrease, mostly due to the dispersion of some cells into the extracellular matrix (Figure 2d). The MCF10A Crβ4 cysts also exhibited a generally spheroid shape, albeit demonstrating a lower circularity index (Figure 2a,b), but their growth was slower compared with the MCF10A CTL cysts (Figure 2c). This could be partly due to a reduced cell proliferation rate. Indeed, loss of Na_V_β4 expression slightly reduced MCF10A cell viability after 5 days of culture (Supplementary Figure S1b), which appeared to be due to a slower DNA synthesis (Supplementary Figure S1c,d). However, it also appeared that cells evaded from the MCF10A Crβ4 cysts to invade the 3D extracellular matrix (Figure 2a, arrows), thus resulting in a complete disorganisation of cysts and in a reduction of the number of individualized cysts with time (Figure 2a,d). We thus characterized the cyst ultrastructure. Cysts were fixed and stained in order to visualise cell nuclei, vimentin expression, and F-actin network by fluorescence imaging. The MCF10A CTL cysts demonstrated a typical and regular epithelial organisation with compacted cell nuclei uniformly distributed within the structure, expression of vimentin at the external periphery of the cyst (equivalent to the basal side of the epithelial layer), contiguous cells displaying a cortical submembrane F-actin network, and the appearance of a central lumen (Figure 2e). By contrast, the MCF10A Crβ4 cysts were completely disorganized, lacked a central lumen, and did not show any apico-basal polarisation (Figure 2e). Cell nuclei appeared to be less compacted, the vimentin expression was more intense and diffuse, and the cortical F-actin network was disrupted to be more diffuse inside the cytosol. Furthermore, multiple cells detached from the cyst to invade the extracellular matrix. Because this characteristic might require proteolytic activities, we explored the capacity of the MCF10A cysts to degrade the fluorogenic substrate DQ-Gelatin when incorporated into the extracellular matrix, after 7 days of growth (Figure 2f). The MCF10A CTL cysts demonstrated an almost undetectable proteolytic activity. By contrast, the MCF10A Crβ4 cysts showed a strong proteolytic activity towards the extracellular matrix (green fluorescence) and a strong invasion by cells originating from the cyst (Figure 2f,g). Because cell–cell junctions appeared to be disrupted in the MCF10A Crβ4 cysts, we then analysed the expression of adherens junction proteins β-catenin and E-cadherin. While the MCF10A CTL cysts were characterized by a strong colocalization of β-catenin and E-cadherin at the cell plasma membrane, the MCF10A Crβ4 cysts demonstrated a weaker and diffuse expression of both proteins with no colocalization (Figure 3a). These observations prompted us to investigate the expression of β-catenin depending on that of Na_V_β4. Consistent with the more mesenchymal phenotype, the mRNA level of gene coding for β-catenin, *CTNNB1*, was significantly higher in the MCF10A Crβ4 compared with MCF10A CTL cells (40% median increase, Figure 3b). In contrast, β-catenin protein level was down-regulated in the MCF10A Crβ4 compared with MCF10A CTL cells (52% median decrease, Figure 3c). A possible explanation for this apparent discrepancy could be an increased recycling of β-catenin in the MCF10A Crβ4 cells. Therefore, we incubated the MCF10A CTL and MCF10A Crβ4 cells with the proteasome inhibitor MG132 for different time durations and assessed levels of β-catenin (Figure 3d). These led to an increased immunodetection of β-catenin in both cell lines. Nevertheless, the increase in β-catenin protein expression was significantly higher in the MCF10A Crβ4 than in MCF10A CTL cells (Figure 3e). Similarly, the MCF10A cells transfected with specific silencing RNA targeting Na_V_β4 (siβ4, Figure 3f) also displayed a reduced expression of β-catenin compared with scramble siRNA (siCTL, Figure 3g), and this expression was restored upon treatment with MG132 (Figure 3h). These results suggested that the loss of the plasma membrane Na_V_β4 led to the disruption of intercellular junctions and to a reduced half-life of β-catenin. Together with the loss of the epithelial morphology in individual cells, the disorganisation and the increased extracellular invasiveness of cysts, the disruption of adherens junctions pointed in favour of a transition towards a mesenchymal-like phenotype. Therefore, we next examined the expression of genes associated with either epithelial phenotype (*CDH1*, coding for E-cadherin) or with mesenchymal phenotype (*CDH2*, coding for N-cadherin; *SNAI1, SNAI2, TWIST, ZEB1* coding for transcription factors promoting epithelial-to-mesenchymal transition, *VIM*, coding for vimentin, and *ACTA2*, coding for α-SMA). The loss of Na_V_β4 expression was associated with a significant reduction in the epithelial marker *CDH1* expression, and an increase in the expression of mesenchymal markers *CDH2*, *SNAI2, TWIST, ZEB1*, *VIM*, and *ACTA2* (Figure 4a). Only the *SNAI1* expression was not modified. These changes observed at the transcriptional level were confirmed at the protein level for E-cadherin, N-cadherin, vimentin, and α-SMA (Figure 4b). Finally, we explored the possibility to rescue the epithelial phenotype in MCF10A Crβ4 by overexpressing Na_V_β4 (Figure 4c). This partially restored the *CDH2*, *VIM*, and *ACTA2* expression (Figure 4d).

## 4. Discussion

Pore-forming Na_V_α and auxiliary Na_V_β proteins of voltage-gated sodium channels were initially characterized in excitable cells in which they are responsible for triggering and propagation of action potentials. However, over the past years, it has been shown that both Na_V_α and Na_V_β subunits are dysregulated in cancers in which they have non-excitable roles. Most of the time, their overexpression in carcinoma cells has been associated with cancer progression, and their activity was shown to promote pro-cancerous properties [39]. Pore-forming Na_V_1.5, Na_V_1.6, and Na_V_1.7 appear to be specifically upregulated in cancer cells and their activity, through an inward sodium current, have been shown to promote invasive properties [40]. It has also been found that some Na_V_β subunits are upregulated in cancer and that they bear important roles in cancer cell biology and in cancer progression [39]. In contrast, the Na_V_β4 subunit has recently been identified as being expressed at high levels in normal epithelial tissues, but consistently downregulated in cancer samples. This was identified in breast, lung, prostate, colorectal [29], cervical [30] and papillary thyroid [31] cancer. In these studies, the preserved expression of the *SCN4B* gene was considered as a favourable biomarker of metastasis-free survival, suggesting that it could be a metastasis-suppressor gene.

The expression of the *SCN4B* gene was shown to be lower in highly invasive cancer as compared with weakly invasive or non-cancer cells [29,30,41]. Both in vitro and in vivo experiments, performed with different human cancer cell lines, demonstrated that reducing the Na_V_β4 expression potentiated cell migration, invasiveness, and tumour progression, while overexpressing it had opposite effects [29,30,42]. In breast cancer cells, the loss of Na_V_β4 promoted RhoA activity and the acquisition of a hybrid mesenchymal–amoeboid phenotype associated with highly invasive capacities [29]. However, the role of Na_V_β4 in normal epithelial cells is not known, and whether its loss might participate in events of early carcinogenesis has never been elucidated.

In this study, we confirmed that *SCN4B*/Na_V_β4 is downregulated in cancer compared with non-cancer cells and tissues. Furthermore, we demonstrated that its repression in non-cancer mammary epithelial cells induced dramatic morphological and functional changes associated with a loss of epithelial polarisation, disruption of epithelial junctions, overexpression of genes associated with a mesenchymal phenotype, and acquisition of pro-invasive capacities. Consequently, the loss of Na_V_β4 completely abrogated the establishment of 3D epithelial structures (cysts). The loss of Na_V_β4 favoured degradation of β-catenin, thus reducing its half-life and leading to the disruption of adherens junctions. All these effects were completely independent on the expression of the pore-forming Na_V_1.5 channel, which is a marker of breast cancer aggressiveness [39]. It can be speculated that the Na_V_β4 subunit, expressed at the plasma membrane of epithelial cells, stabilizes β-catenin in proximity to E-cadherin. The loss of Na_V_β4 might lead to cytosolic release of β-catenin part of which could translocate to the nucleus and induce the expression of EMT-related genes, and a second part could undergo proteasomal degradation. These cellular events might be critical during carcinogenesis.

However, what could trigger the repression of *SCN4B* during cancer transformation is still unknown. Interestingly, recent studies pointed out the involvement of several miRNAs that are dysregulated in some cancers as important regulators of the *SCN4B* expression. In colorectal cancer, the increased expression of miR-424-5p in tumour samples was associated with poor prognosis [43]. In this study, the authors demonstrated that *SCN4B* was directly inhibited by miR-424-5p, thus promoting colon cancer cell proliferation, migration, and invasion [43]. Another miRNA, miR-3175, has recently been shown to be overexpressed in prostate cancer and to participate in cancer cell growth and invasion. Knocking down miR-3175 in prostate cancer cells increased the *SCN4B* and E-cadherin expression, inhibited the N-cadherin expression, and importantly reduced cell proliferation, migration, and invasion [44]. Together, these recent studies brought new elements on the involvement of miRNA in the promotion of cancer progression through the regulation of the *SCN4B* expression in tumours. As such, exploring the expression of these miRNAs during carcinogenesis might be of importance.

In conclusion, this study demonstrated the critical role played by Na_V_β4 in the maintenance of an epithelial phenotype in normal cells, and how its repression might contribute to cellular dysplasia and early carcinogenesis. In this context, maintaining its expression level in epithelial cells would be determinant in order to prevent, or delay, tumour transformation.

## Data Availability

All supporting data are available upon request.

## Supplementary Materials

The following are available online at https://www.mdpi.com/article/10.3390/cells10071624/s1, Figure S1: Viability and proliferation of MCF10A CTL and MCF10A Crβ4 cells, Figure S2: uncropped WB films shown in, Table S1: Primers used for PCR.
