# Supplementary material for: The Voltage-Gated Sodium Channel Beta4 Subunit Maintains Epithelial Phenotype in Mammary Cells
Source: Cells. 2021 Jun 29;10(7):1624. doi: 10.3390/cells10071624 (PMC8304757; doi:10.3390/cells10071624)
Supplement: Supplementary file 1 [file cells-10-01624-s001.zip › Doray et al, Supplementary figures new.pdf]

Supplementary Figure 1

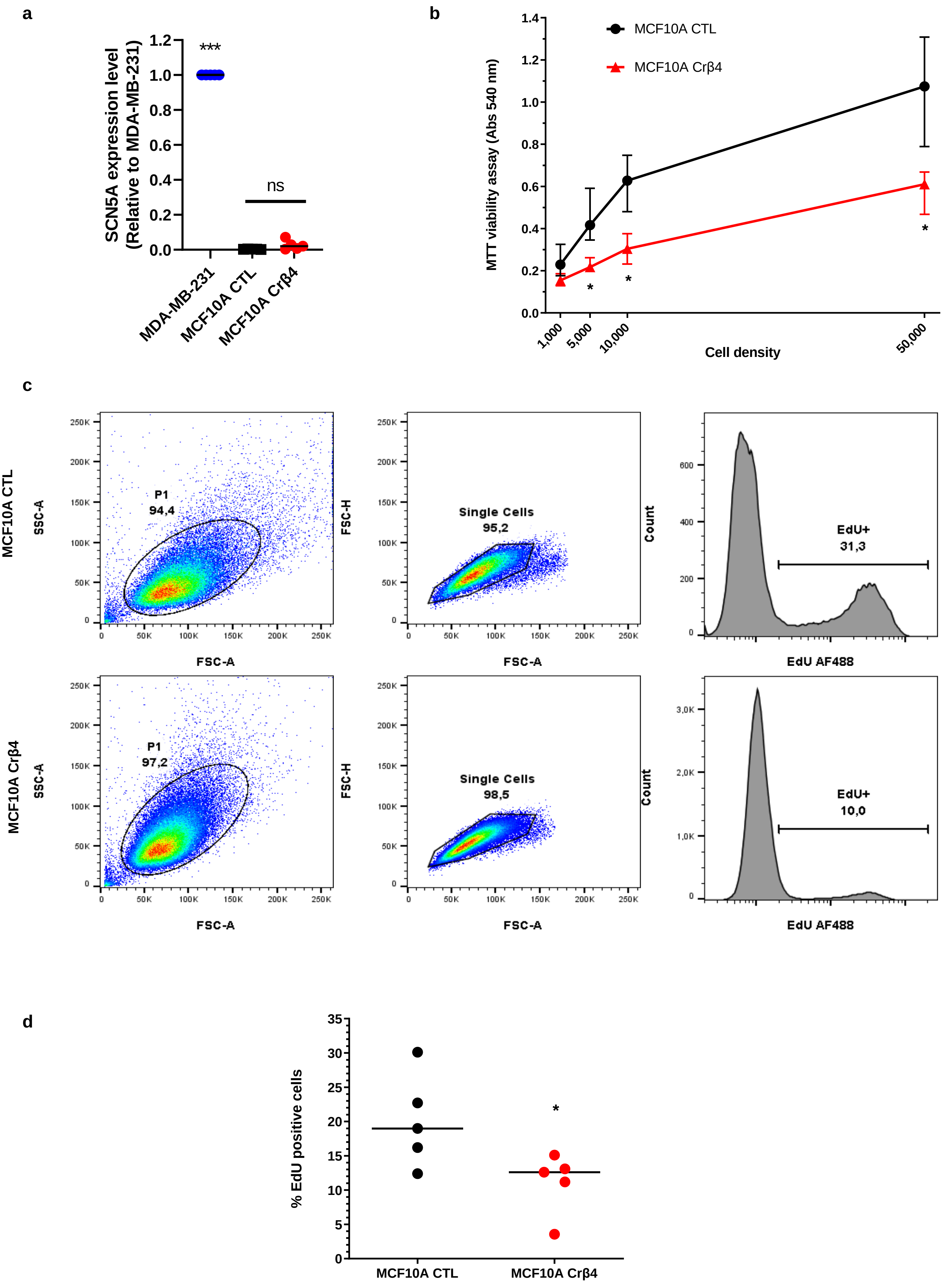

Supplementary Figure 2

a) Uncropped blots shown in figure 1a

Na$_V$β4

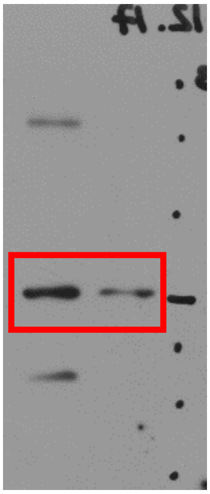

HSC70

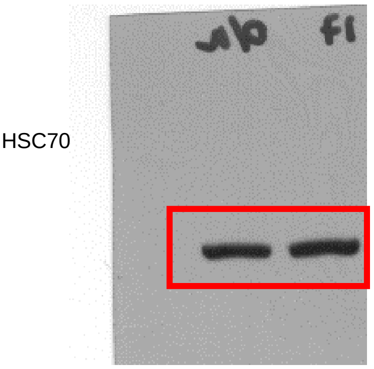

b) Uncropped blots shown in figure 1b

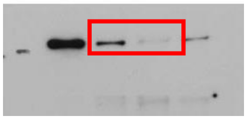

Na$_V$β4

HSC70

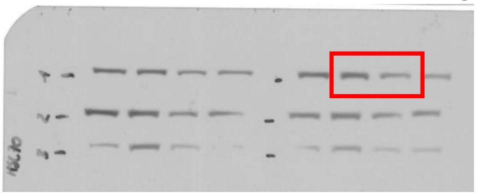

c) Uncropped blots shown in figure 3c

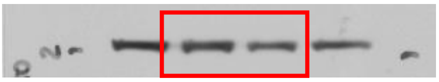

HSC70

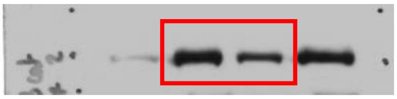

B-catenin

d) Uncropped blots shown in figure 3d

B-actin

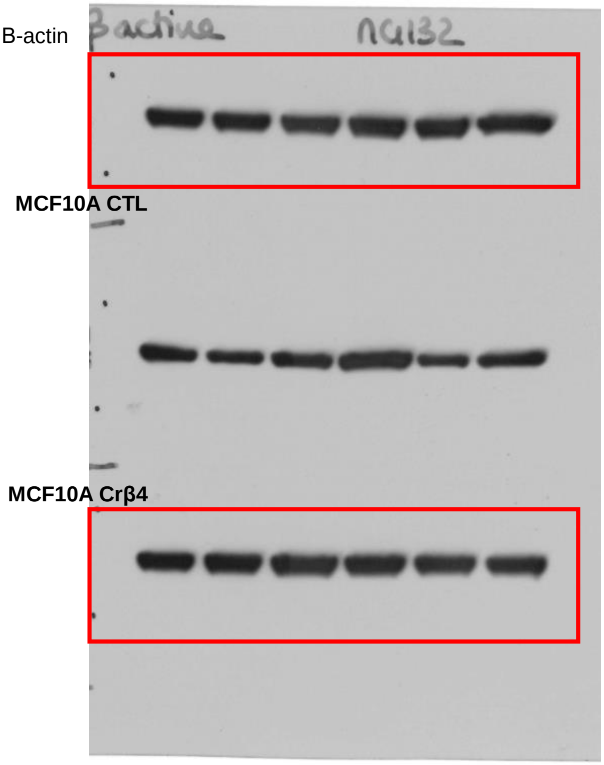

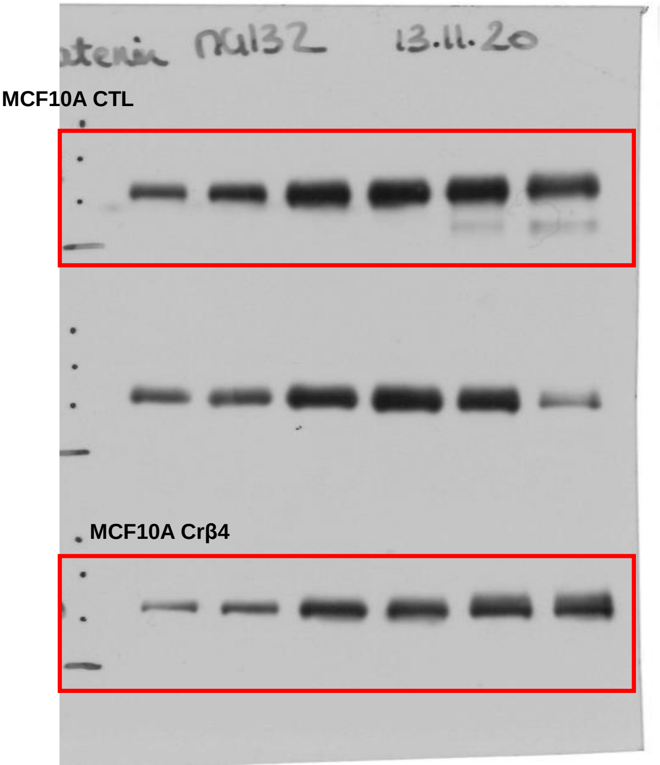

B-catenin

e) Uncropped blots shown in figure 3f

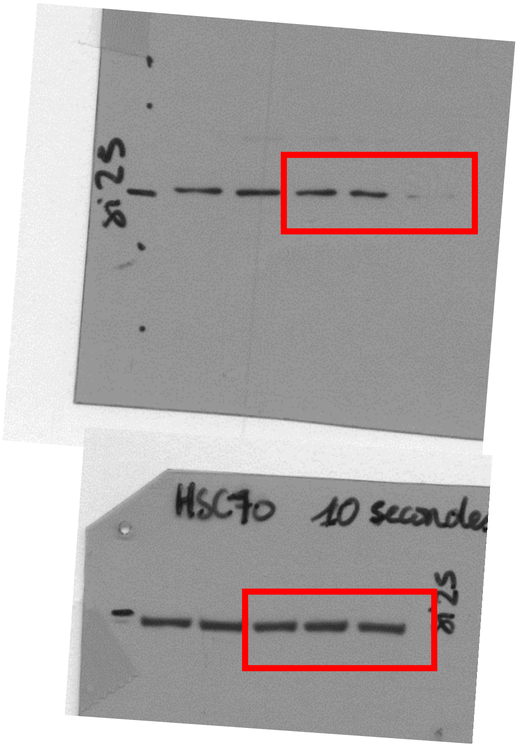

f) Uncropped blots shown in figure 3g

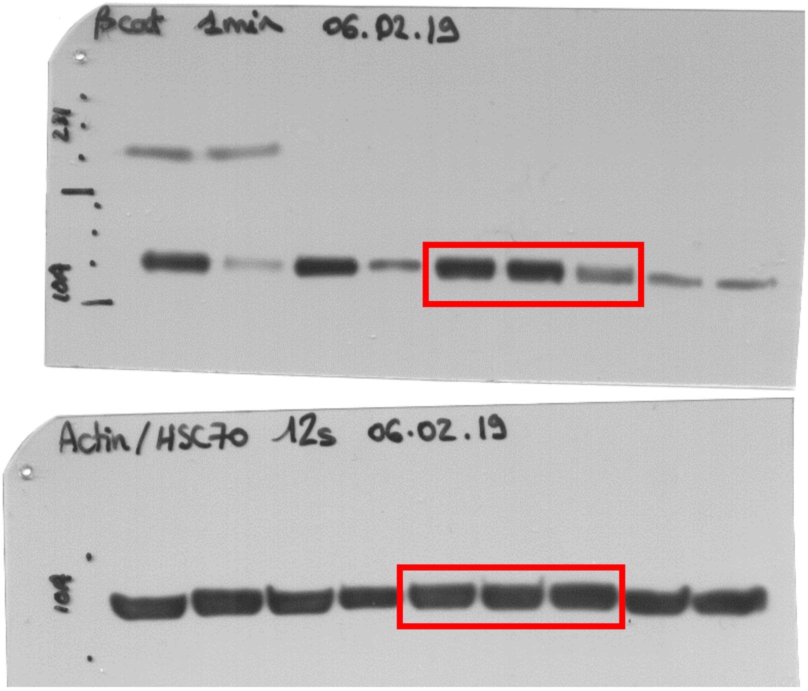

g) Uncropped blots shown in figure 3h

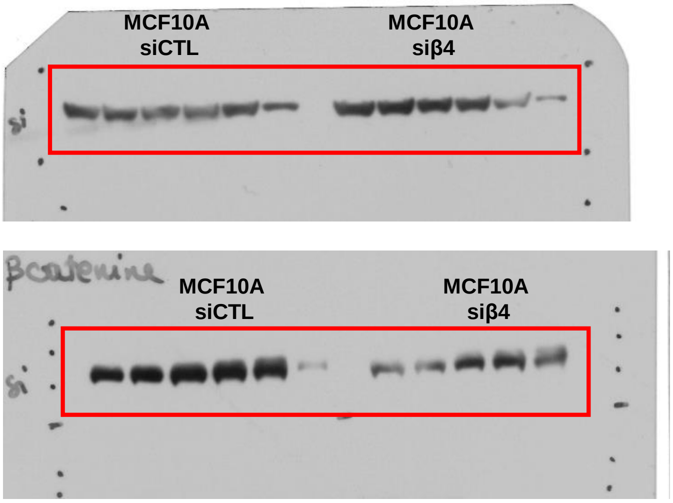

h) Uncropped blots shown in figure 4b

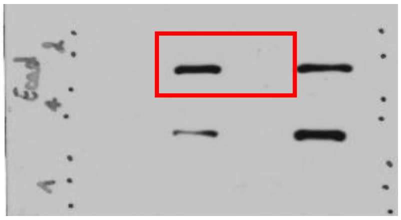

E-cadherin

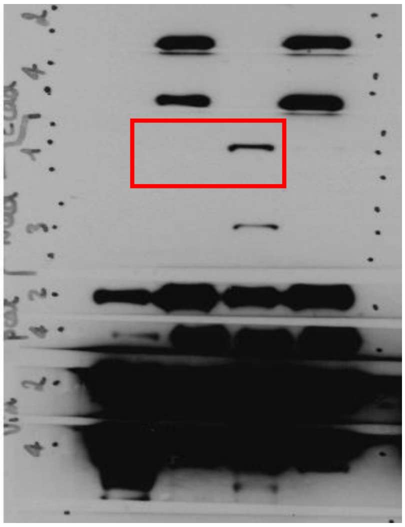

N-cadherin

Vimentin

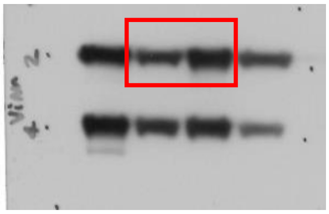

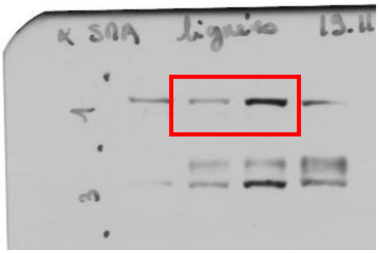

αSMA

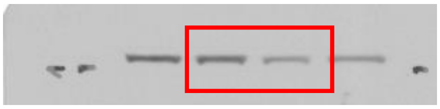

HSC70

h) Uncropped blots shown in figure 4c

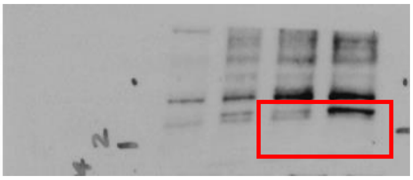

$Na_V\beta 4$

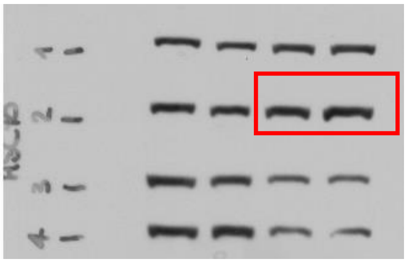

HSC70
