# Supplementary material for: The Voltage-Gated Sodium Channel Beta4 Subunit Maintains Epithelial Phenotype in Mammary Cells
Source: Cells. 2021 Jun 29;10(7):1624. doi: 10.3390/cells10071624 (PMC8304757; doi:10.3390/cells10071624)
Supplement: Supplementary file 1 [file cells-10-01624-s001.zip › Doray et al, Supplementary materials new.pdf]

Supplementary materials

THE VOLTAGE-GATED SODIUM CHANNEL BETA4 SUBUNIT MAINTAINS EPITHELIAL PHENOTYPE IN MAMMARY CELLS

Adélaïde DORAY[1], Roxane LEMOINE[1], Marc SEVERIN[2], Stéphanie CHADET[1], Osbaldo LOPEZ-CHARCAS[1], Audrey HÉRAUD[1], Christophe BARON[1], Pierre BESSON[1], Arnaud MONTEIL[3], Stine Falsig PEDERSEN[2] & Sébastien ROGER[1,4§]

[1] University of Tours, EA4245 Transplantation, Immunology, Inflammation, Tours, France

[2] Section for Cell Biology and Physiology, Department of Biology, Faculty of Science, University of Copenhagen, Copenhagen, Denmark

[3] IGF, University of Montpellier, CNRS, INSERM, Montpellier, France

[4] Institut Universitaire de France, Paris, France

[§]Correspondence: Dr. Sébastien Roger,

EA4245 Transplantation, Immunology, Inflammation, 10 Bd Tonnellé, 37032 Tours, France
Tel: (+33) 2 47 36 61 30, Email: sebastien.roger@univ-tours.fr

Supplementary Figure 1: Viability and proliferation of MCF10A CTL and MCF10A Crβ4 cells

a, The expression of the *SCN5A* gene, encoding for $Na_V1.5$ was monitored by RT-qPCR in MDA-MB-231 breast cancer cells, in MCF10A CTL and MCF10A Crβ4 mammary non-cancer cells. (n=5 independent experiments). ***, p<0.001 as compared to MCF10A CTL or MCF10A Crβ4. Ns, stands for no statistical difference between MCF10A CTL and MCF10A Crβ4. **b**, MCF10A CTL and MCF10A Crβ4 cell viability was measured by the MTT assay 4 days after seeding the cells at different densities (n=6 independent experiments). *, p<0.05 (Wilcoxon test). **c**, Labelling with 10 µM 5-Ethynyl-2'deoxyuridine (EdU, 10 µM) in MCF10A CTL and MCF10A Crβ4 cells was assessed by flow cytometry after 3 days of culture. **d**, Analyses of results acquired as in (b) from 5 independent experiments. *, p<0.05 (Mann-Whitney rank sum test).

Supplementary Figure 2: uncropped WB films shown in a, Figure 1b, **b**, Figure 1c, **c,** Figure 3c, **d,** Figure 3d**, e,** Figure 3f**, f,** Figure 3g**, g,** Figure 3h**, h,** Figure 4b**,** and **f,** Figure 4c**.**

Supplementary Figure 1

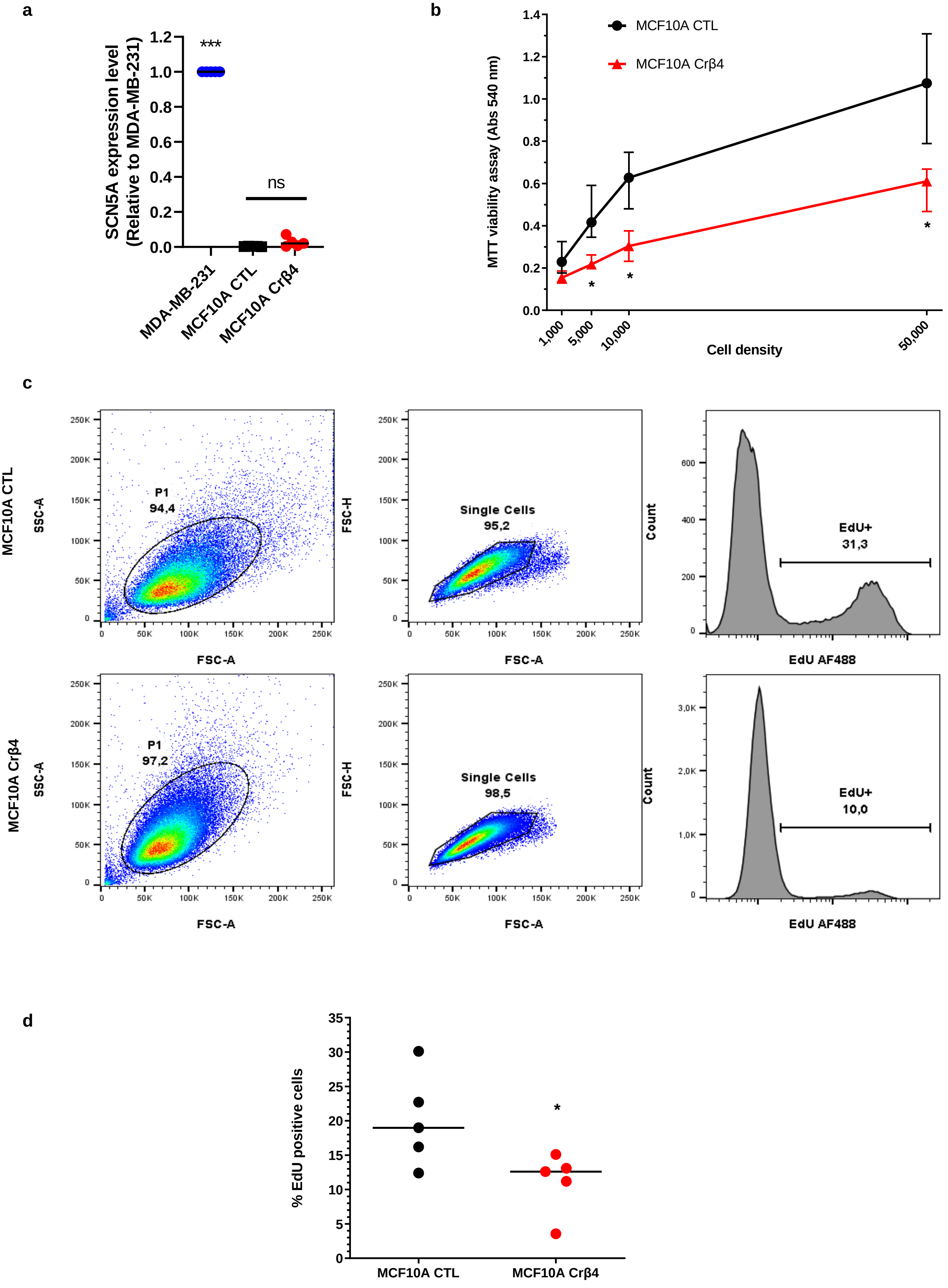

Supplementary Figure 2

a) Uncropped blots shown in figure 1a

$Na_V\beta4$

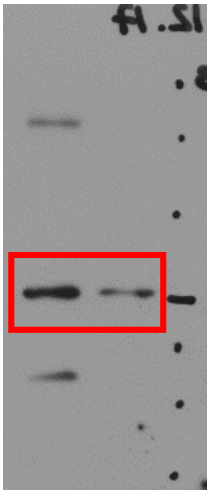

HSC70

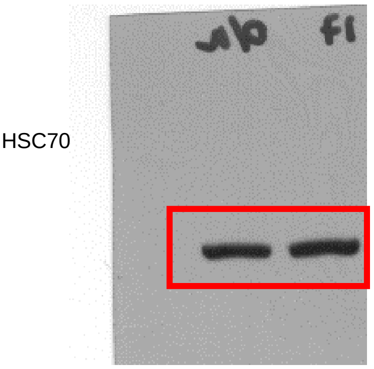

b) Uncropped blots shown in figure 1b

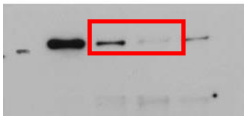

$Na_V\beta4$

HSC70

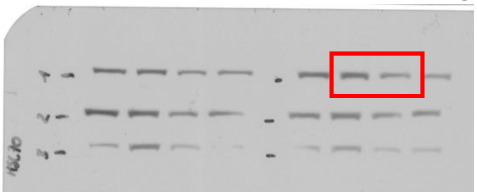

c) Uncropped blots shown in figure 3c

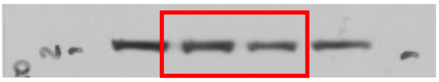

HSC70

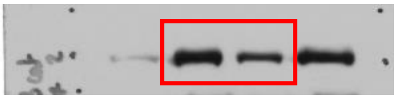

B-catenin

d) Uncropped blots shown in figure 3d

B-actin

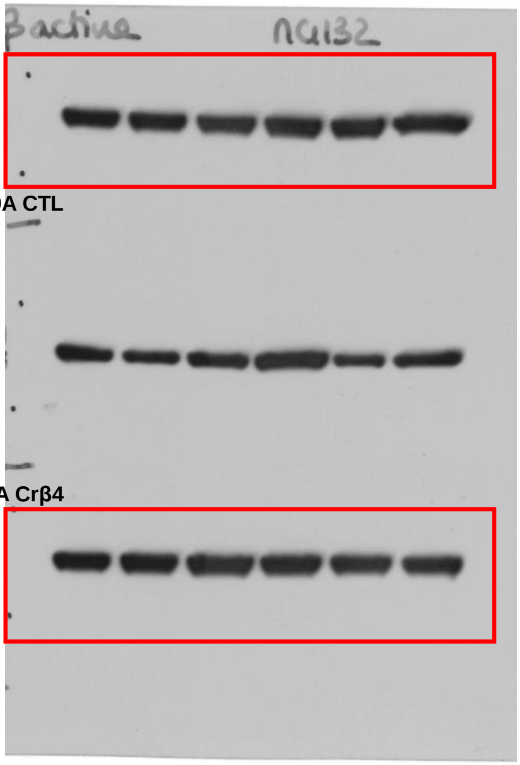

MCF10A CTL

MCF10A Crβ4

B-catenin

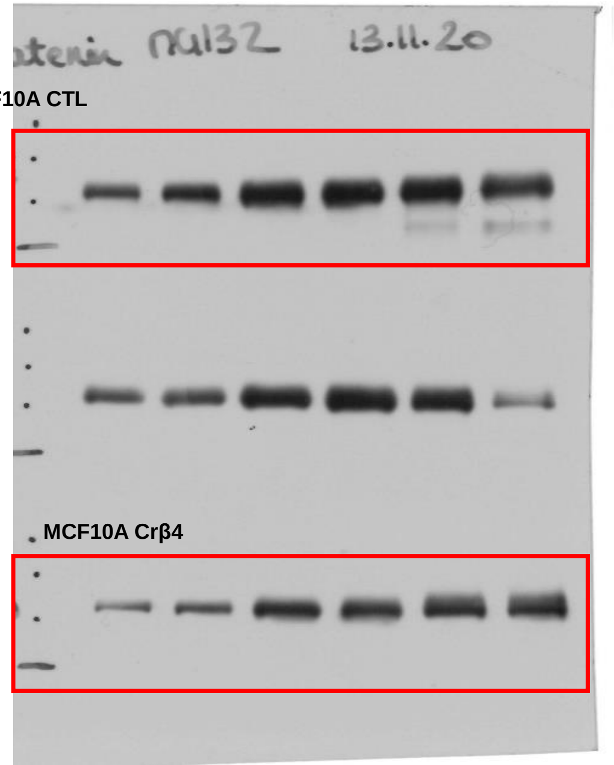

MCF10A CTL

MCF10A Crβ4

e) Uncropped blots shown in figure 3f

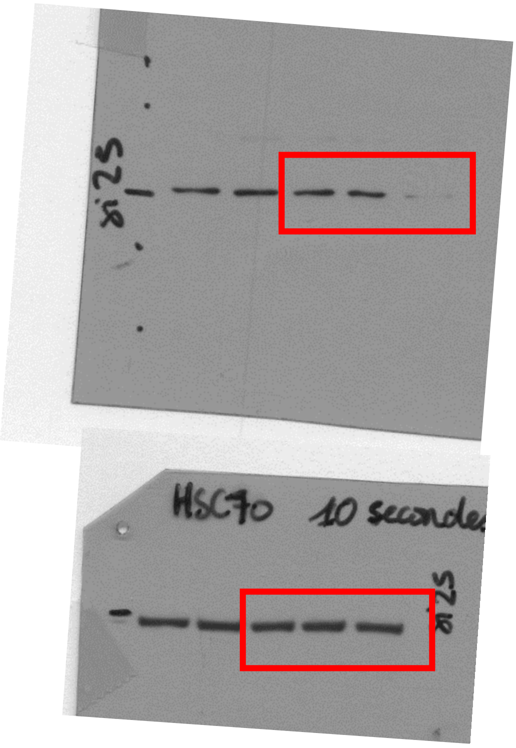

f) Uncropped blots shown in figure 3g

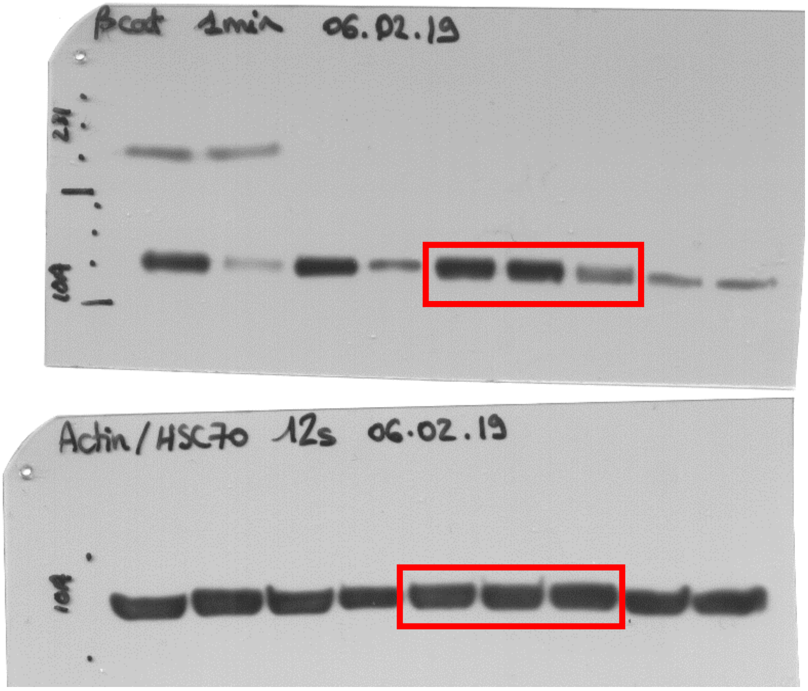

g) Uncropped blots shown in figure 3h

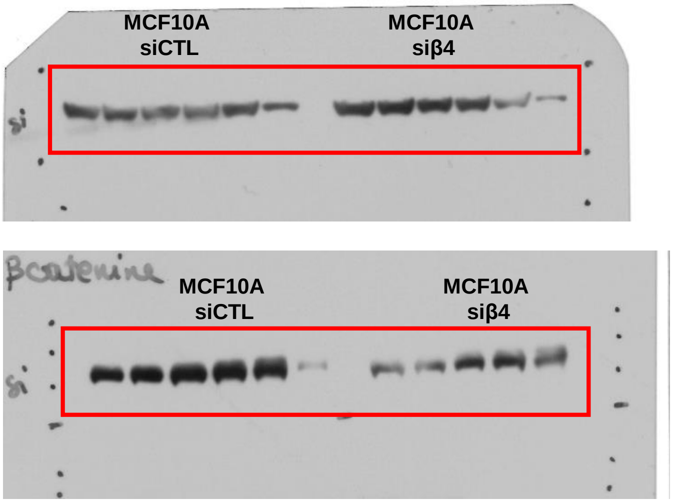

h) Uncropped blots shown in figure 4b

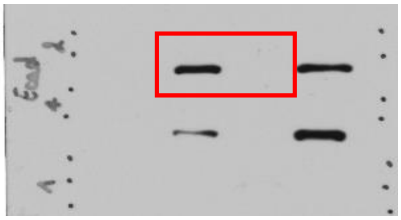

E-cadherin

N-cadherin

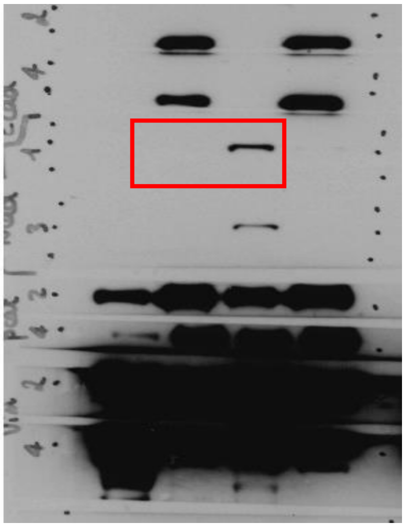

Vimentin

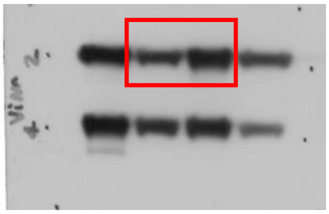

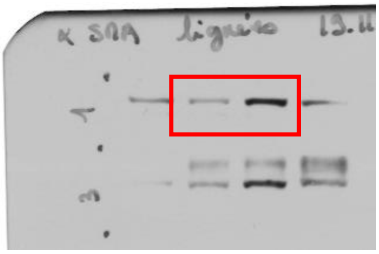

αSMA

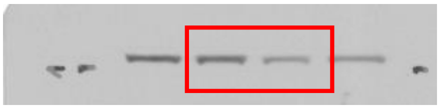

HSC70

h) Uncropped blots shown in figure 4c

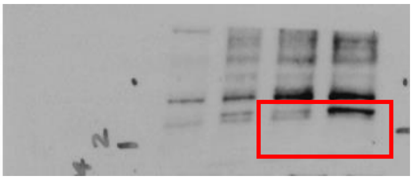

$Na_V\beta4$

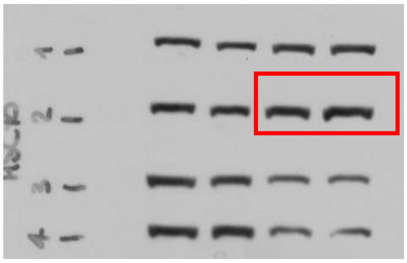

HSC70

Supplementary Table I: PCR primers used

**Genes**	**Proteins**	**Forward primer (5’→3’)**	**Reverse primer (5’ →3’)**	**Efficiencies**	**Expected size (pb)**
CTNNB1	β-catenin	CCCACTAATGTCCAGCGTTT	GCATGATAGCGTGTCTGGAA	2.00	214
CDH1	E-cadherin	CGACCCAACCCAAGAATCTA	GGCTGTGCCTTCCTACAGAC	2.03	171
CDH2	N-cadherin	GGTGGAGGAGAAGAAGACCAG	GGCATCAGGCTCCACAGT	2.09	72
SNAI1	Snail	GGTTCTTCTGCGCTACTGCT	TAGGGCTGCTGGAAGGTAAA	2.00	157
SNAI2	Slug	GAGCATTTGCAGACAGGTCA	GCTTCGGAGTGAAGAAATGC	2.10	200
TWIST1	Twist1	CCACTGAAAGGAAAGGCATC	GCATTTTACCATGGGTCCTC	2.01	229
ZEB1	Zeb1	GCACCTGAAGAGGACCAGAG	GTGTAACTGCACAGGGAGCA	2.08	200
VIM	Vimentin	GTTTCCAAGCCTGACCTCAC	TTCCAGGGACTCATTGGTTC	1.99	246
ACTA2	α-SMA	ACCCGATAGAACATGGCATC	CATACATGGCTGGGACATTG	2.03	195
SCN5A	$Na_V1.5$	CACGCGTTCACTTTCCTTC	CACGCGTTCACTTTCCTTC	2.00	208
HPRT1	Hprt1	TTGCTGACCTGCTGGATTAC	TATGTCCCCTGTTGACTGGT	2.00	119
