# Supplementary material for: The Voltage-Gated Sodium Channel Beta4 Subunit Maintains Epithelial Phenotype in Mammary Cells
Source: Cells. 2021 Jun 29;10(7):1624. doi: 10.3390/cells10071624 (PMC8304757; doi:10.3390/cells10071624)
Supplement: Supplementary file 1 [file cells-10-01624-s001.zip › Doray et al., Suppl. table I new.pdf]

Supplementary Table I: PCR primers used

**Genes**	**Proteins**	**Forward primer (5'→3')**	**Reverse primer (5' →3')**	**Efficiencies**	**Expected size (pb)**
CTNNB1	β-catenin	CCCACTAATGTCCAGCGTTT	GCATGATAGCGTGTCTGGAA	2.00	214
CDH1	E-cadherin	CGACCCAACCCAAGAATCTA	GGCTGTGCCTTCCTACAGAC	2.03	171
CDH2	N-cadherin	GGTGGAGGAGAAGAAGACCAG	GGCATCAGGCTCCACAGT	2.09	72
SNAI1	Snail	GGTTCTTCTGCGCTACTGCT	TAGGGCTGCTGGAAGGTAAA	2.00	157
SNAI2	Slug	GAGCATTTGCAGACAGGTCA	GCTTCGGAGTGAAGAAATGC	2.10	200
TWIST1	Twist1	CCACTGAAAGGAAAGGCATC	GCATTTTACCATGGGTCCTC	2.01	229
ZEB1	Zeb1	GCACCTGAAGAGGACCAGAG	GTGTAACTGCACAGGGAGCA	2.08	200
VIM	Vimentin	GTTTCCAAGCCTGACCTCAC	TTCCAGGGACTCATTGGTTC	1.99	246
ACTA2	α-SMA	ACCCGATAGAACATGGCATC	CATACATGGCTGGGACATTG	2.03	195
SCN5A	$Na_V1.5$	CACGCGTTCACTTTCCTTC	CACGCGTTCACTTTCCTTC	2.00	208
HPRT1	Hprt1	TTGCTGACCTGCTGGATTAC	TATGTCCCCTGTTGACTGGT	2.00	119
