# Supplementary material for: The Voltage-Gated Sodium Channel Beta4 Subunit Maintains Epithelial Phenotype in Mammary Cells
Source: Cells. 2021 Jun 29;10(7):1624. doi: 10.3390/cells10071624 (PMC8304757; doi:10.3390/cells10071624)
Supplement: Supplementary file 1 [file cells-10-01624-s001.zip › Doray et al., Supplementary figure legends_revised.pdf]

Supplementary materials

THE VOLTAGE-GATED SODIUM CHANNEL BETA4 SUBUNIT MAINTAINS EPITHELIAL PHENOTYPE IN MAMMARY CELLS

Adélaïde DORAY[1], Roxane LEMOINE[1], Marc SEVERIN[2], Stéphanie CHADET[1], Osbaldo LOPEZ-CHARCAS[1], Audrey HÉRAUD[1], Christophe BARON[1], Pierre BESSON[1], Arnaud MONTEIL[3], Stine Falsig PEDERSEN[2] & Sébastien ROGER[1,4§]

[1] University of Tours, EA4245 Transplantation, Immunology, Inflammation, Tours, France

[2] Section for Cell Biology and Physiology, Department of Biology, Faculty of Science, University of Copenhagen, Copenhagen, Denmark

[3] IGF, University of Montpellier, CNRS, INSERM, Montpellier, France

[4] Institut Universitaire de France, Paris, France

[§]Correspondence: Dr. Sébastien Roger,

EA4245 Transplantation, Immunology, Inflammation, 10 Bd Tonnellé, 37032 Tours, France
Tel: (+33) 2 47 36 61 30, Email: sebastien.roger@univ-tours.fr

Supplementary Figure 1: Viability and proliferation of MCF10A CTL and MCF10A Crβ4 cells

a, The expression of the *SCN5A* gene, encoding for Nav1.5 was monitored by RT-qPCR in MDA-MB-231 breast cancer cells, in MCF10A CTL and MCF10A Crβ4 mammary non-cancer cells. (n=5 independent experiments). ***, $p<0.001$ as compared to MCF10A CTL or MCF10A Crβ4. Ns, stands for no statistical difference between MCF10A CTL and MCF10A Crβ4. **b**, MCF10A CTL and MCF10A Crβ4 cell viability was measured by the MTT assay 4 days after seeding the cells at different densities (n=6 independent experiments). *, $p<0.05$ (Wilcoxon test). **c**, Labelling with 10 µM 5-Ethynyl-2'deoxyuridine (EdU, 10 µM) in MCF10A CTL and MCF10A Crβ4 cells was assessed by flow cytometry after 3 days of culture. **d**, Analyses of results acquired as in (b) from 5 independent experiments. *, $p<0.05$ (Mann-Whitney rank sum test).

Supplementary Figure 2: uncropped WB films shown in a, Figure 1b, **b**, Figure 1c, **c,** Figure 3c, **d,** Figure 3d**, e,** Figure 3f**, f,** Figure 3g**, g,** Figure 3h**, h,** Figure 4b**,** and **f,** Figure 4c**.**
